# Single nucleotide polymorphisms of the IL28B and sustained virologic response of patients with chronic hepatitis C to PEG-interferon/ribavirin therapy: A meta-analysis: Meta-analysis of IL28B

**Published:** 2011-03-01

**Authors:** Shiying Li, Peng Hu, Qin-Qin Zhang, Ying-Hong Liu, Huai-Dong Hu, Da-Zhi Zhang, Hong Ren

**Affiliations:** 1 Department of Infectious Diseases, Institute for Viral Hepatitis, Key Laboratory of Molecular Biology for Infectious Diseases, Ministry of Education, The Second Affiliated Hospital of Chongqing Medical University, Chongqing, China

**Keywords:** Chronic hepatitis C, Single nucleotide polymophisms, Interleukin 28B, human, Antiviral agents

## Abstract

**Background:**

Hepatitis C is a global health problem and represents a major cause of liver disease and socioeconomic burden. Effective antiviral therapy may prevent these complications, but the current treatment for patients with chronic hepatitis C virus (HCV) infection does not produce sustained virologic response. Therefore, identification of the determinants of response to treatment is a high priority. A number of host and viral factors have been associated with treatment outcomes.

**Objectives:**

To assess the associations of single nucleotide polymorphisms (SNP) of the IL28B and sustained virologic response (SVR) of patients with chronic hepatitis C to PEG-interferon/ribavirin therapy.

**Materials and Methods:**

We searched PubMed, Medline and Cochrane Library, and found 7 eligible papers involved in this study. Then we performed a meta-analysis comparing the SVR rate at SNP of the IL28B in individuals with PEG-interferon/ribavirin therapy. Meanwhile, the SVR rate between different races and HCV genotypes was studied.

**Results:**

The sustained virologic response rate was higher in patients with the rs12979860 CC and rs8099917 TT alleles in the IL28B SNP, comparing with the rs12979860 CT, or TT and rs8099917 TG or GG. Furthermore, a higher SVR was observed in the Caucasians than in Afro-Americans (OR = 3.85, 95% CI: 3.06-4.83); the percentage of rs12979860 TT genotype was lower in Caucasians (OR = 0.25, 95% CI: 0.20-0.31) and the percentage of rs12979860 CC genotype was higher in Caucasians than that of Afro-Americans (OR = 3.45, 95% CI = 2.68-4.44). Between different HCV genotypes, the SVR was much lower in those with HCV genotype 1 than those with genotype 2/3 (OR = 0.16, 95% CI: 0.11-0.24).

**Conclusions:**

IL28B is significantly associated with response to PEG-interferon/ribavirin therapy of patients with chronic HCV infection. Both the rs12979860 and rs8099917 alleles could be used as independent predictors of the treatment response. The rs12979860 allele in particular, is more important from our study. The polymorphism even explains part the difference in response rate between different ethnic groups and HCV genotypes.

## Background

Hepatitis C is a global health problem and represents a major cause of liver disease and socioeconomic burden. There were 120-180 million hepatitis C virus (HCV) carriers worldwide, with worldwide prevalence estimated at 3% [1][2][3], and a new 3-4 million cases appearing each year [2]. Only a minority of these infected patients spontaneously clear HCV. Failed to clear, 70%-80% of patients become chronic carriers [4], who may progress to liver cirrhosis and hepatocellular carcinoma (HCC) leading to the need of liver transplantation [5][6][7]. Effective antiviral therapy may prevent these complications, but the current treatment for patients with chronic HCV infection, a combination of pegylated interferon-α 2a or 2b (PEG-IFN-α) given by injection with oral ribavirin (RBV), does not produce sustained virologic response (SVR) in all patients treated. This treatment is not only long and costly, but also associated with signiﬁcant side effects (e.g., a flu-like syndrome, hematologic abnormalities and adverse neuropsychiatric events) [8], resulting in reduced compliance and fewer patients completing the treatment. For these reasons, identification of the determinants of response to treatment is a high priority. A number of host and viral factors have been associated with treatment outcomes [9][10][11][12].

The viral factors, including HCV genotype, baseline viral load, viral kinetics during treatment, and amino acid pattern in the interferon sensitivity-determining region, have been fully studied [13][14][15]. HCV genotype, in particular, is used in making treatment decisions: patients with HCV genotype 2/3 have a relatively high rate of SVR (70%-80%) with 24 weeks of treatment, whereas those infected with HCV genotype 1 have a much lower rate of SVR (40%-52%) despite 48 weeks of treatment [16][17][18][19]. The host factors include age, sex, body mass index (BMI), insulin resistance, hepatic steatosis, hepatic ﬁbrosis and ethnicity [9][12][18][20][21][22]. Recently, several highly correlated single nucleotide polymorphisms (SNP) on a linkage disequilibrium block in the vicinity of 3 IFN-λ genes on chromosome 19, encoding INF-λ1 (IL29), λ2 (IL28A), and λ3 (IL28B), have been implicated in response to PEG-IFN/RBV among patients infected with HCV from four studies [23][24][25][26], which also seems to explain part of the difference in response between different races [23] and HCV genotypes [26]. Even with a lot of researches in this field, there is a controversy about it.

## Objectives

To clarify the role of rs12979860 and rs8099917 alleles, which were researched the most of IL28B, in treatment response of patients with chronic hepatitis C (CHC) to PEG-IFN/RBV, we carried out a meta-analysis of the available cohort studies of the association between the SNP of IL28B and the SVR of patients with CHC treated with PEG-IFN/RBV.

## Materials and Methods

### Selection criteria

Analytic epidemiological studies (cross-sectional, independent replication cohort, or genome-wide association study) that examined the SNP of IL28B in response to PEG-IFN/RBV among patients infected with HCV were reviewed. Attempts were made to contact the authors where data was missing, by e-mail. The following studies were included in the review:

1. Articles in English only were included in the review.

2. Observational epidemiological studies were included.

3. Articles were limited to humans only.

4. Conference reports.

### Exclusion criteria

1. Case reports.

2. Case series.

3. Studies not limited to humans.

4. Studies not in English.

5. Studies that did not provide enough information to calculate the treatment effects.

### Literature search

**Figure 1:**
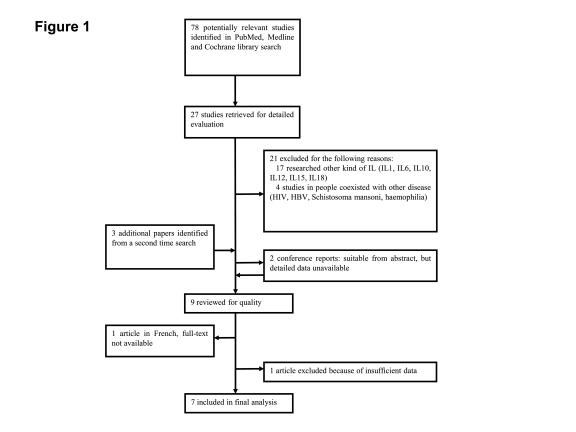
Flow diagram of literature search

We identiﬁed the studies by searching PubMed, Medline and Cochrane Library with the following search terms: ("IL"[MeSH] OR "interleukin"[MeSH]) AND ("INF"[MeSH] OR "interferon"[MeSH]) AND ("hepatitis B"[MeSH] OR "HBV"[MeSH] OR "CHB"[MeSH] OR "hepatitis C"[MeSH] OR "HCV"[MeSH]) AND ("SNP" [MeSH] OR "polymorphism"[MeSH] OR "mutation"[MeSH]) AND "humans"[MeSH]. The initial search done on May 20, 2010, retrieved 78 articles. The abstracts of these 78 papers were read by two reviewers independently. After screening with both the selection criteria and exclusion criteria, only 27 studies were found eligible for further evaluation. Then 21 papers were excluded for not meeting the inclusion criteria with a deeper review. Three additional studies from a second time search, done on June 24, 2010, were also included. In the nine left papers, one was excluded because of insufficient data [27], another article was in French, and its full-text was not available [28]. And, two conference reports from the 2010 ASDL found to be eligible based on their abstracts, but the detailed data were not available after contacting their authors. Thus, in the final analysis, seven articles [23][24][25][26][29][30][31]were included (Figure 1), with a total number of 4791 patients studied.

### Data extraction

A full detailed description of each study cohort is presented in Table 1. Study subjects from Switzerland were came from the Swiss Hepatitis C Cohort Study and the Swiss HIV Cohort Study, two multicenter studies performed at eight major Swiss hospitals and their local afﬁliated centers [32][33], and from the Medical Clinic for Hepatology and Gastroenterology, Medical University Charité Campus, Virchow-Klinikum Berlin, in Berlin, Germany. A genome-wide association study of sustained virological response (SVR) to PEG-IFN-α/RBV combination therapy with genotype 1 chronic hepatitis C was performed in three studies from USA [9]and Australia, disparate an additional replication cohort of white people from the Australian, and a further prospective treatment study from one of the USA [12]. Another study from USA was based on a cross-sectional study. And, the subjects from Japan were patients with CHC at the Graduate School of Medicine, Kanazawa University Hospital and its related hospitals.

**Table 1:** Descriptive baseline characteristics of the subjects included in this study

**Study**	**Sample size**	**Race**	**Genotype**	**Drug**	**Therapy time**	**Follow-up time**	**Efficacy measures**
**Ge D, et al. **(2009)	1137	946 (Caucasians)	1137 (1)	PEG-IFN-α-2a or -2b/RBV	48 wk	24w	SVR, non-SVR
		191 (Afro-Americans)					
**Suppiah V, et al . **(2009)	848	848 (Caucasians)	848 (1)	PEG-INF-α/RBV	48 wk	24w	SVR, NR
**Tanaka Y, et al. **(2009)	314	314 (Asians)	314 (1)	PEG-INF-α/RBV	48 wk	24w	SVR, VR, NR
**Honda M, et al. **(2009)	168	168 (Asians)	168 (1)	PEG-INF-α-2b/RVB	48 wk	24w	SVR, TR, NR, EVR
**McCarthy JJ, et al. **(2009)	231	178 (Caucasians)	186 (1)	NA	NA	24w	SVR, NR, Relapsers
		53 (Afro-Americans)	45 (2/3)				
**Rauch A, et al. **(2009)	465	465 (Caucasians)	188 (1)	PEG-INF-α/RBV	48 wk	24w	SVR, NR
			222 (2/3)				
			34 (4)				
			21 (others)				
**Thompson AJ, et al. ** (2009)	1628	1287 (Caucasians)	1628 (1)	PEG-INF/RBV	48 wk	24w	SVR, EVR, RVR
		300 (Afro-Americans)					
		41 (Others)					

### Definition of main concepts

SVR was defined as "having undetectable HCV-RNA levels 24 weeks after cessation of treatment." Non-responders were patients whose HCV-RNA levels remained detectable at the end of treatment. Those who had undetectable levels of HCV-RNA at the end of treatment, but detectable HCV-RNA levels at 24 weeks after cessation of treatment were called "relapsers." Both the non-responders and relapsers were categorized as non-viral response (NVR). Rapid viral response (RVR) was with an undetectable HCV-RNA levels at four weeks and early viral response (EVR) was at 12 weeks.

### Statistical analysis

The main outcome of interest was odds ratios (OR), which estimated the association between SNP of IL28B and the treatment response. Crude association estimates and 95% confidence intervals (95% CI) from each study were calculated, and displayed by forest plots. In the forest plot, the relative weight of each study's contribution to the analysis was represented by the area of box whose center represents the estimated measure of association. Both the fixed effect model and random effects model were used in this meta-analysis. The random effects model provides a way to address the heterogeneous studies which varied in their design, characteristics, and sampled populations and other factors contributing to variation in the data. The estimated confidence interval will be wider than that from a fixed effect model if there is heterogeneity among the studies and, thus, protects against falsely assuming a significant difference when there is none. Random effects model is used when the Q statistic obtained is significant which indicates that there is variability among the effect sizes which may be attributed to factors or sources which may not be identifiable or measurable. But the fixed effect model is chosen if the Q statistic obtained is not significant. All analyses were done with Review Manager ver 4.2.2.

## Results

### Characteristics of studies

A total of 4791 patients were assessed for this study. A detailed description of each study cohort was presented in Table 1. Three of the world main ethnicities were all included. The Caucasians, Hispanics and the Australian population of northern European ancestry, which was mentioned only in one study, were all typed into the Caucasian group. Others were named as Afro-Americans and Asians in our study. All the studies included were carried out in populations with mean age ranging from 43.4 to 57.4 years. There was a preponderance of males in all studies but one ranging from 54.5% to 64.9% of the subjects. Other detailed information included the HCV genotype, baseline HCVAb level, treatment history and METAVIR fibrosis stage.

### The association between SNP of IL28B (rs12979860) and SVR of CHC patients with PEG-INF/RBV therapy

**Figure 2:**
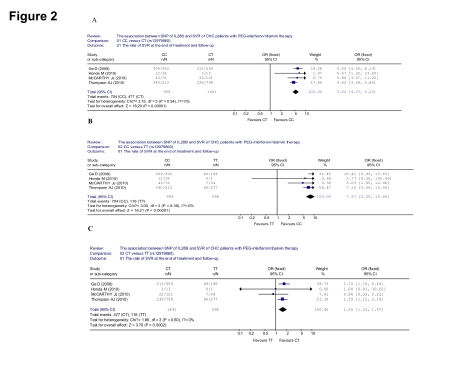
Analysis of the association between SNP of IL28B (rs12979860) and SVR of patients with chronic hepatitis C treated with PEG-INF/RBV. A: Compared the SVR between CC and CT genotypes; B: Compared the SVR between CC and TT genotypes; C: Compared the SVR between CT and TT genotypes

In the seven included papers, only four studied the rs12979860 alleles. We first included this four studies to evaluate the association between SNP of IL28B (rs12979860) and SVR of CHC patients with PEG-INF/RBV therapy. Comparing with the patients of rs12979860 CT genotype, greater SVR was observed in the patients of rs12979860 CC in the four analyzed trials, (704/998 [70.5%] vs 477/1491 [32.0%], OR = 5.22, 95% CI: 4.37-6.23, p < 0.001) (Figure 2A). When comparing the CC and TT genotype, greater SVR was observed in the patients of rs12979860 CC (704/998 [70.5%] vs 116/498 [23.3%], OR = 7.97, 95% CI: 6.20-10.25, p < 0.001) (Figure 2B). When it came to the CT and TT genotype, greater SVR was observed in the patients of rs12979860 CT (447/1491 [32.0%] vs 116/498 [23.3%], OR = 1.56, 95% CI: 1.23-1.97, p < 0.001) (Figure 2C).

### The association between SNP of IL28B (rs8099917) and SVR of CHC patients with PEG-INF/RBV therapy

**Figure 3:**
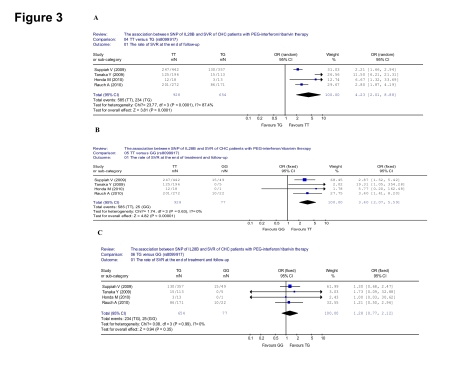
Analysis of the association between SNP of IL28B (rs8099917) and SVR of patients with chronic hepatitis C treated with PEG-INF/RBV therapy. A: Compared the SVR between TT and TG genotypes; B: Compared the SVR between TT and GG genotypes; C: Compared the SVR between TG and GG genotypes

There were four papers studied the rs8099917 alleles in the seven included articles. The evaluation of the association between SNP of IL28B (rs8099917) and SVR of CHC patients with PEG-INF/RBV therapy were done based on these four studies. Greater SVR was observed in the patients of rs8099917 TT, comparing with the patients of rs8099917 TG genotype (585/928 [63.0%] vs 234/654 [35.8%], OR = 4.23, 95% CI: 2.01-8.88, p < 0.001) (Figure 3A). When focusing the TT and GG genotype, greater SVR was observed in the patients of rs8099917 TT (585/928 [63.0%] vs 25/77 [32.5%], OR = 3.40, 95% CI: 2.07-5.59, p < 0.001) (Figure 3B). But between the TG and GG genotype, the difference was not significant (234/654 [35.8%] vs 25/77 [32.5%], OR = 1.28, 95% CI: 0.77-2.12, p = 0.35) (Figure 3C)

### The association between the SNP of IL28B (rs12979860) and SVR of chronic hepatitis C patients with PEG-INF/RBV therapy by race

**Figure 4:**
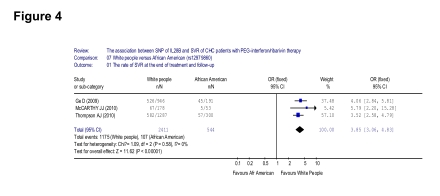
Analysis of the association between the rate of SVR and ethnicity

Only three of the seven included papers studied both the Caucasians and Afro-Americans, despite three of the world main ethnicities were all included. And, they were all discussed the rs12979860 alleles only. From these three papers, a higher SVR was observed in the Caucasians than Afro-Americans (1175/2411 [48.7%] vs 107/544 [20.0%], OR = 3.85, 95% CI: 3.06-4.83, p < 0.001) (Figure 4).

**Figure 5:**
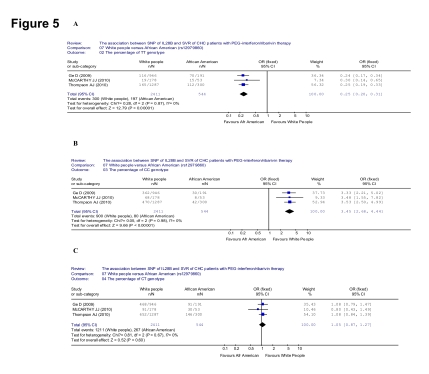
Analysis of the association between the percentage of each genotype of rs12979860 and ethnicity. A: Compared the percentage of TT genotype; B: Compared the percentage of CC genotype; C: Compared the percentage of CT genotype

Then, we searched the association between the percentage of each genotype of rs12979860 and people ethnicities. The percentage of TT genotype was lower in the Caucasians than that of Afro-Americans (300/2411 [12.4%] vs 197/544 [36.2%], OR = 0.25, 95% CI: 0.20-0.31, p < 0.001) (Figure 5A). The percentage of CC genotype was higher in the Caucasians than that of Afro-Americans (900/2411 [37.3%] vs 80/544 [14.7%], OR = 3.45, 95% CI: 2.68-4.44, p < 0.001) (Figure 5B). When it came to CT genotype, however, the percentage was not significant between the Caucasians and Afro-Americans (1211/2411 [50.2%] vs 267/544 [49.1%], OR = 1.05, 95% CI: 0.87-1.27, p < 0.001) (Figure 5C).

### The association between the SNP of IL28B (rs12979860 and rs8099917) and SVR of chronic hepatitis C patients with PEG-INF/RBV therapy by HCV genotype

**Figure 6:**
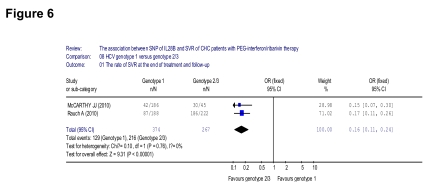
Analysis of the association between the rate of SVR and HCV genotype

In the seven included papers, only two investigated different HCV genotypes. We grouped them into the genotype 1 and genotype 2/3 group because the genotypes 2 and 3 cannot be separated clearly according to one of this initial study. About the IL28B, one was focused on the rs12979860 allele, while another was focused on rs8099917. First, we did a meta-analysis of the association between the rate of SVR and HCV genotype. A lower SVR was observed in the HCV genotype 1 group, comparing with genotype 2/3 (129/347 [37.2%] vs 216/267 [80.9%], OR = 0.16, 95% CI: 0.11-0.24, p < 0.001) (Figure 6).

**Figure 7:**
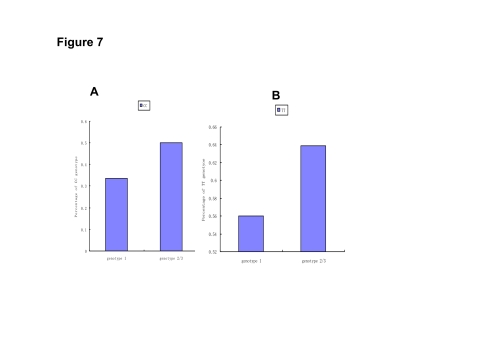
Association between IL28B and HCV genotype. A: The rs12979860 CC percentage in different HCV genotypes (by the study of McCarthy JJ, et al); B: The rs8099917 TT percentage in different HCV genotypes (by the study of Rauch A, et al)

Then the two different loci of IL28B were analyzed separately. The percentage of rs12979860 CC was much higher in HCV genotype 2/3 (50.0%) than that of genotype 1 (33.5%, p < 0.001). But the percentage of rs8099917 TT among HCV genotype 2/3 (63.9%) and genotype 1 (55.6%, p = 0.07) was not much different (Figure 7).

## Discussion

Multiple viral and host factors may be related to the treatment response to PEG-IFN/RBV combination therapy for the CHC patients. For the host factors, the hepatic INF-stimulated genes (ISGs), such as Myxovirus resistance 1 (Mx1), 2'-5'-oligoadenylate synthetase 3 (OAS3), INF-induced protein 44 (IFI44), INF-induced protein 44-like (IFI44L), 2'-5'-oligoadenylate synthetase 2 (OAS2), ubiquitin specific peptidase 18 (USP18), radical S-adenosyl methionine domain containing 2 (RSAD2), INF-induced with tetratricopeptide repeats 1 (IFIT1), INF-induced with helicase C domain 1 (IFIH1), XIAP associated factor-1 (XAF1), cytidine monophosphate kinase 2 (CMPK2), epithelial stromal interaction 1 (breast) (EPSTI1), hect domain and RLD 6 (HERC6), poly (ADP-ribose) polymerase family, member 9 (PARP9), and phospholipid scramblase 1 (PLSCR1) were all reported earlier. But the interesting phenomenon was that the IL28B genotype was demonstrated to be a strongly independent predictor of the treatment response for the CHC patients in a multivariate logistic regression analysis among many other host and viral factors, nearly in all the studies included. The role of IL28B was reported a lot recently, which was also thought to be associated with spontaneous HCV clearance [34], and a signiﬁcant independent predictor of response to PEG-IFN/RBV in patients with chronic HCV infection [26]. From Dongliang Ge, et al, the genetic polymorphism near the IL28B gene (rs12979860) was associated with an approximately two-fold change in response to treatment, both among patients of European ancestry and Afro-Americans. And, the greater frequency of the genotype leading to better response (rs12979860 CC alleles) in European than African populations, also explains approximately half of the difference in response rates between Afro-Americans and patients of European ancestry [23]. Other studies emphasize that the major effect of this polymorphism (rs12979860) was to increase the rate of early viral decline, leading to higher SVR rates [30]. Moreover, Masao H, et al. [29], demonstrated that expression of INF-stimulated genes (ISGs), a new reported determinant viral factor, was related to genetic variation in IL28B. To the contrary, two SNPs near the gene IL28B on chromosome 19 (rs12980275 and rs8099917) were found to be strongly associated with NVR.

The protein product of IL-28B is IFN-λ-3, one of the three members of the recently described type III IFN family (IFN-λ-1/2/3 = IL-29, IL-28A, and IL-28B) [35][36], which have been studied previously in the context of HBV and HCV infection and shown to suppress both HBV and HCV replication [37][38][39]. In co-stimulation experiments, IFN-λ and IFN-α have an additive antiviral effect. But the mechanisms through which IL-28B variant genotypes inﬂuence antiviral response to PEG-IFN-α/RBV remain unclear. Some researches believe in a major role of the innate immunity in the control of HCV. IFN-λ interacts with a transmembrane receptor to induce potent antiviral responses [35][36].This antiviral activity is mediated through the activation of the J AK-STAT (IFN-α, IFN-λ, and IFN-λ) and MAPK (IFN-α and IFN-λ) pathways. In vitro and in vivo models have shown the importance of IFN-λ in the immune response to several viral pathogens, including herpes simplex virus [40][41],cytomegalovirus [42], HIV [43], hepatitis B and C virus [44]. IFN-λ1 and IFN-λ2 block HCV replication in human hepatocytic cell lines [37][38][39]. Other studied explained the antiviral activity by mediating the ligand for TLR3 and TRL9, an antiviral protection [39][44].

The mechanism of rs12979860 allele, which located 3 kb upstream of the IL28 gene, and rs8099917, locating 8.9 kb from the end of transcription of IL28B and 16 kb from the end of transcription of IL28A, related to PEG-IFN-α/RBV response is not yet known. It is likely that the activity or levels of the nearby IFN-λ genes is influenced, because responders to treatment are characterized as having a lower baseline immune response to HCV [45][46]. This could also explain the paradoxical association of the response genotype with higher viral load in the study by Ge, et al [22]. In this meta-analysis, either the rs12979860 CC genotype or rs8099917 TT genotype received a much better response of therapy, regardless of comparing with the rs12979860 CT genotype/rs8099917 TG genotype or the rs12979860 TT genotype/rs8099917 GG genotype. For this reason, the results supported that the C base at 12979860 and the T base at rs8099917 were the advantaged bases leading to better response to the combination therapy of PEG-INF/RBV. And, the difference was higher in the major homozygote (rs12979860 CC/rs8099917 TT) and heterozygote, compared with the heterozygote and the minor homozygote (rs12979860 TT/rs8099917 GG), which indicated a more important role of the risk base (rs12979860 T/rs8099917 G) in the treatment effect than that of protective base (rs12979860 C/rs8099917 T). But the rs12979860 seemed to be more related to a better response.

The patients of European ancestry had a significantly higher probability of being cured than patients of African ancestry. The percentage of rs12979860 CC in the Caucasians was much higher than that of Afro-Americans, fitting the much lower percentage of rs12979860 TT in the Caucasians than that of Afro-Americans. This proved that the C base at 12979860 was the advantaged base leading to better response to the combination therapy of PEG-INF/RBV again and at the same time the T base at 12979860 was a risk factor for treatment from another aspect. It also explains part of the reason that the difference of therapy response in different ethnicities may be due to the different occurrence of rs12979860 genotypes. Similarly, the HCV genotypes 2/3 had a much greater SVR than that of genotype 1. The percentage of rs12979860 CC in the HCV genotypes 2/3 group was higher than that of genotype 1, but the difference of rs8099917 TT percentage in the HCV genotypes 2/3 group and genotype 1 was not much significant. It seemed to explain part of the reason that the difference of therapy response in different HCV genotypes may be related to the SNP of IL28B. But the current data was not suitable for a meta-analysis.

Furthermore, the role of baseline HCV-RNA, liver fibrosis stage, and drug type in the SVR of PEG-INF/RBV therapy was not clear according to the present included data. We concluded that the polymorphism of IL28B is significantly associated with response to PEG-IFN/RBV therapy for patients with chronic HCV infection. Rs12979860, in particular, could be used as an independent predictor of the treatment response. And, the polymorphism explains part the difference in response between different ethnicities and HCV genotypes. Further studies are needed to explore the mechanism of the reported genetic association at IL28B, and the association of this polymorphism and other treatment response impacting factors such as the baseline HCV-RNA, gender and age.
